# Morphological variation in atlas and axis of Neotropical spiny rats (Rodentia, Echimyidae)

**DOI:** 10.1002/ar.70087

**Published:** 2025-11-15

**Authors:** Thomas Furtado da Silva Netto, João Felipe Leal Kaiuca, Adriana Itatí Olivares, William Corrêa Tavares

**Affiliations:** ^1^ Programa de Pós‐Graduação em Biodiversidade e Biologia Evolutiva, Instituto de Biologia Universidade Federal do Rio de Janeiro Rio de Janeiro Brazil; ^2^ Núcleo Multidisciplinar de Pesquisa em Biologia, Campus UFRJ Duque de Caxias Professor Geraldo Cidade Universidade Federal do Rio de Janeiro, Duque de Caxias Rio de Janeiro Brazil; ^3^ Laboratório de Tafonomia e Sistemática de Vertebrados Fósseis, Departamento de Geologia e Paleontologia, Museu Nacional Universidade Federal do Rio de Janeiro Rio de Janeiro Brazil; ^4^ Sección Mastozoología, División Zoología Vertebrados, Museo de La Plata, Facultad de Ciencias Naturales y Museo de La Plata Universidad Nacional de La Plata, CONICET La Plata, Buenos Aires Argentina

**Keywords:** Caviomorpha, cervical vertebrae, geometric morphometrics, locomotion, Octodontoidea

## Abstract

The unique morphologies of the first two cervical vertebrae, the atlas and axis, represent a significant innovation in mammalian evolution. These structures support the weight of the head and enable intricate movements of the head and neck. Within Caviomorpha, Echimyidae (spiny rats, coypu, and hutias) exhibit considerable variation in vertebral size and encompass arboreal, terrestrial, semi‐fossorial, and semi‐aquatic lifestyles, making them a promising model for investigating how vertebral morphology responds to different ecological pressures. We analyzed 99 atlas and 76 axis vertebrae from 23 extant species and the extinct *Eumysops chapalmalensis*, using two‐dimensional geometric morphometrics combined with phylogenetic comparative methods. Linear discriminant analyses were used to infer the locomotor mode of the extinct species. Our results reveal that the morphology of the atlas and axis is shaped by phylogenetic relatedness, vertebral size (allometry), and locomotor modes. Although allometry significantly influenced vertebral shape, its overall effect was limited compared to the stronger role of locomotor specialization. We detected significant associations between vertebral shape and both vertebral size and ecological specialization in most analyzed views. Although each vertebra contributes to identifying the major echimyid clades and their locomotor strategies, the atlas exhibits the strongest overall signal. We also describe morphofunctional adaptations related to head stabilization and movement. Our findings support the hypothesis that *E. chapalmalensis* was a terrestrial or semi‐fossorial rodent. Altogether, the results underscore the value of axial skeletal elements, particularly the atlas and axis, in revealing patterns of adaptive specialization in both extant and extinct species.

## 1 INTRODUCTION

The axial skeleton of vertebrates has multiple functions, including body and head support, balance, locomotion and lung ventilation (Arnold, 2021; Jones et al., 2018a; Verbout, 1985). In the lineage that originated mammals, the vertebral column evolved a unique functional regionalization, more evident in eutherians, resulting in five main modules based on developmental and morphological identity: cervical, thoracic, lumbar, sacral and caudal regions (e.g., Jones et al., 2018a). This vertebral regionalization favored the radiation of mammals in a wide variety of locomotor modes (Jones et al., 2018a). Among the various regions of the mammalian spine, most comparative studies have focused on the thoracic and lumbar regions. These studies reveal significant biomechanical specializations linked with locomotor modes, as well as allometric constraints and phylogenetic effects (e.g., Granatosky et al., 2014; Hautier et al., 2010; Jones et al., 2018a; Netto & Tavares, 2021; Shapiro, 1995; Shapiro & Simons, 2002). In contrast, the factors influencing the morphology of cervical vertebrae remain poorly understood, with most studies focusing only on carnivores and primates (Manfreda et al., 2006; Martins et al., 2021; Randau et al., 2016), and seldom exploring other mammalian groups (Gaudioso et al., 2017; Hatt, 1932; Vander Linden, Campbell, et al., 2019; Vander Linden, Hedrick, et al., 2019).

The mammalian cervical vertebrae are a highly conservative serial structure regarding the number of constituent elements. All mammals have seven cervical vertebrae (Buchholtz & Stepien, 2009; Hautier et al., 2010; Varela‐Lasheras et al., 2011), with the exception of manatees (*Trichechus*) and tree sloths (*Choloepus* and *Bradypus*). Although neck length varies substantially among some mammalian clades (e.g., giraffes and whales), the “rule of seven” is still conserved (Müller et al., 2021). Despite the strict control over the number of cervical vertebrae (Galis et al., 2006, 2018), notable morphological variation exists among them. In mammals, the cervical region exhibits its own regionalization, with cranial/upper cervical (C1–C2), intermediate cervicals (C3–C5), and caudal/lower cervical (C6–C7) forming distinct modules (Arnold et al., 2016; Randau & Goswami, 2017; Villamil, 2018). Cervical vertebrae support the weight of the head, allow complex movements of the head and neck, and house the spinal cord (e.g., Evans, 1939; Kemp, 1969, 2005). The atlas (C1) and axis (C2) have unique shapes, differing from the other cervical vertebrae. The atlas‐axis complex in mammals is morphologically more specialized than in other tetrapods, enabling greater head mobility and representing significant innovations in mammalian evolution (Evans, 1939). In addition, each of them provides attachment for muscles responsible for different movements. The atlas articulates anteriorly with the occipital condyles of the cranium and posteriorly with the odontoid process of the axis, allowing flexion and extension of the head, and head rotation, respectively (Evans, 1939; Kemp, 1969, 2005; Vidal et al., 1988). The axis, on the other hand, articulates with the caudal portion of the atlas and the cranial portion of C3, enabling lateral movements of the head. Considering that different strategies for using environmental resources might have different demands for head and neck movements, it is reasonable to hypothesize that among mammalian species different locomotor modes are associated with different morphofunctional specializations of the atlas and axis (Vander Linden, Campbell, et al., 2019).

The Echimyidae, consisting of the Neotropical spiny rats, the coypu, and the hutias, is the most taxonomically and ecologically diverse family among South American hystricomorph rodents (i.e., caviomorphs). This family comprises at least 102 living species in 28 genera grouped in four subfamilies: Carterodontinae, Capromyinae, Euryzygomatomyinae and Echimyinae; the latter divided into the tribes Echimyini and Myocastorini (Courcelle et al., 2019; Emmons et al., 2015; Emmons & Fabre, 2018; Lacher et al., 2016). Echimyidae currently occupy a wide range of ecosystems, including rainforests, dry forests, savannas, swamps and grasslands from Central America to southern South America (Bonvicino et al., 2008; Carvalho & Salles, 2004; Courcelle et al., 2019; Emmons et al., 2015). Several distinct locomotor modes have been identified for living taxa in this family, including terrestrial, arboreal, semi‐aquatic and semi‐fossorial (e.g., Courcelle et al., 2019; Galewski et al., 2005). The high diversity of locomotor modes among living echimyids was shown to be associated with morphofunctional specializations in different skeletal structures, including scapula, humerus, femur and pelvis (Carvalhaes et al., 2022; Morgan, 2015; Morgan & Álvarez, 2013; Tavares et al., 2020; Tavares & Pessôa, 2020). However, it remains unclear whether the cervical vertebrae in Echimyidae have evolved morphological specializations associated with distinct locomotor modes. In this case of adaptive diversification, significant morphological differences among locomotor groups are expected, independent of body size variation and phylogenetic relatedness. Moreover, these differences should be accompanied by signatures of evolutionary regimes with multiple adaptive optima linked to the divergent locomotor habits within the family (Beaulieu et al., 2012).

Echimyidae is an ancient clade that likely originated between the late Oligocene and the early Miocene (Álvarez et al., 2017; Upham & Patterson, 2015; Verzi et al., 2015, 2018). The fossil record of Echimyidae is poor compared to its current diversity, with less than 10 extinct genera from deposits essentially distributed in southern South America (e.g., Candela et al., 2020; Olivares et al., 2012, 2017; Olivares & Verzi, 2015; Peralta et al., 2025; Piñero et al., 2021; Verzi et al., 2018). Among these extinct genera, *Eumysops*, from the Plio‐Pleistocene of Argentina, is particularly noteworthy due to its exceptional record, characterized by both abundance and excellent preservation. Approximately 90% of its skeleton (including skull, axial and appendicular elements) is known, and its brain morphology has recently been described (Fernández Villoldo et al., 2025; Olivares, 2009; Olivares & Verzi, 2015). Studies based on forelimb and hindlimb morphology suggest that *Eumysops chapalmalensis* was a cursorial echimyid with agile movements or facultative saltatorial capabilities (Horovitz, 1991; Olivares, 2009). In addition, this species has unique craniomandibular and endocranial traits associated with the occupation of open environments, which distinguishes it from extant echimyids (Fernández Villoldo et al., 2025; Olivares et al., 2020).

Due to the remarkable phylogenetic diversity of echimyids and their wide variation in body size and locomotion, this study aims to investigate whether the morphology of the atlas and axis in Echimyidae is associated with phylogenetic, allometric, and ecological factors. We hypothesize that different biomechanical demands associated with locomotor modes shaped the atlas and axis morphology along echimyid diversification. Alternatively, due to the strict and conservative genetic mechanisms acting on the cervical morphogenesis, we also hypothesize that the morphometric variation found in the atlas and axis may reflect phylogenetic structure. In this study, we evaluate the shape variation of the atlas and axis in echimyids using geometric morphometrics, testing those two hypotheses with extant species. We compare evolutionary models to evaluate whether models with multiple adaptive optima associated with distinct locomotor modes better explain extant morphological variation than neutral, non‐adaptive models. We expect that these findings provide insights into how natural selection may have shaped morphological divergence in those vertebrae. Additionally, we incorporated the extinct *Eumysops chapalmalensis* into these analyses to evaluate whether the lifestyle inferred from its atlas and axis aligns with that of the other aforementioned osteological elements. If cervical morphology in echimyids reflects locomotor adaptations in terms of substrate utilization (sensu Eisenberg, 1981: pp. 247–249), we expect to be able to infer the locomotor modes of the extinct species.

## 2 MATERIALS AND METHODS

### 2.1 Sample

**FIGURE 1:**
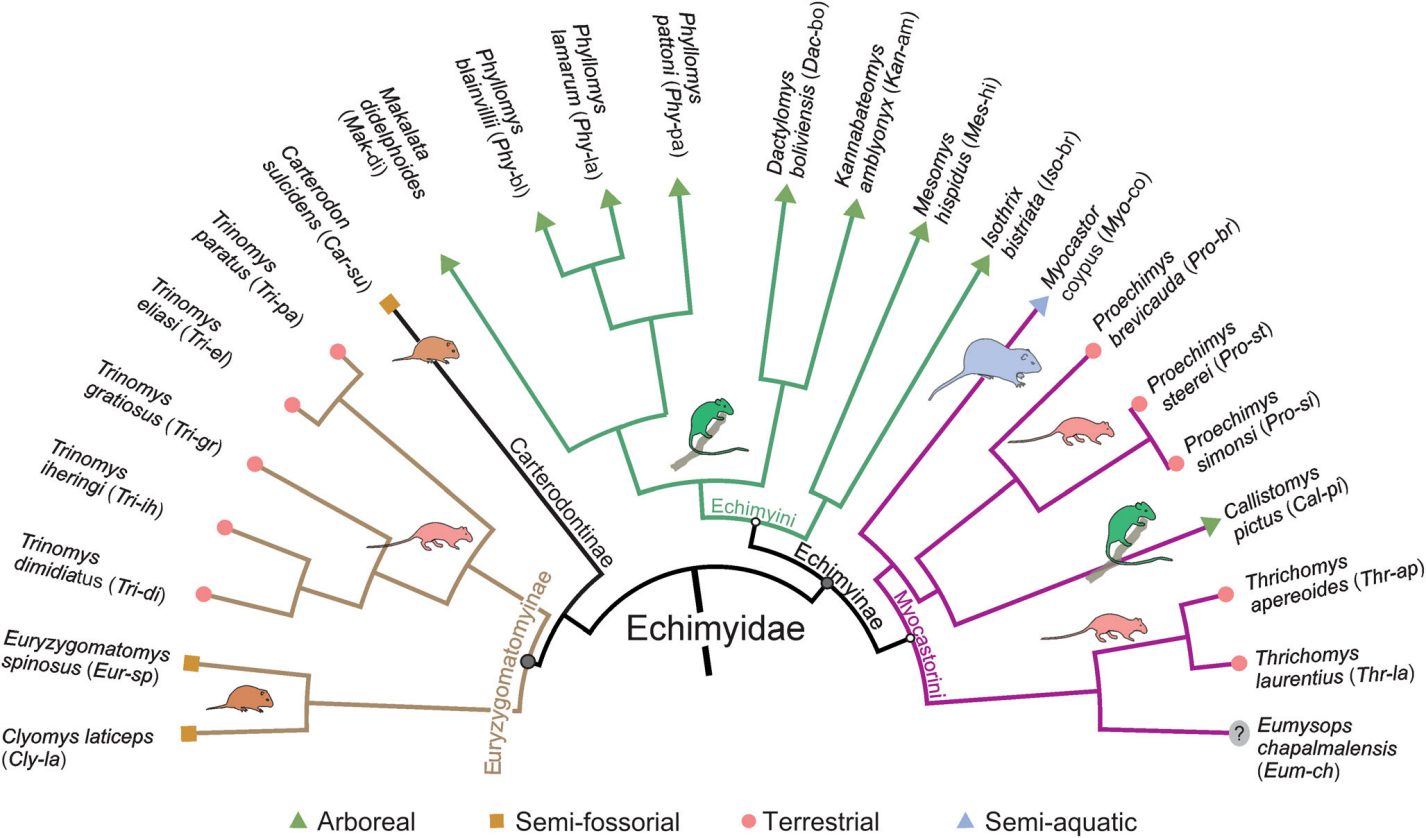
Phylogeny of the species included in the comparative analysis, highlighting locomotor modes and main clades.

Our sample consists of 99 echimyid specimens, including 23 extant and 1 extinct species (*E. chapalmalensis*), 15 genera, and four major lineages: Carterodontinae, Euryzygomatomyinae, Echimyini and Myocastorini (Figure 1). To avoid possible ontogenetic variation, we included in our sample only adult individuals (with all molars erupted, exhibiting signs of wear, and atlas vertebrae with completely fused sutures). However, no distinction was made between male and female individuals, assuming that sexual variation in the cervical vertebrae is negligible and that sexual dimorphism is virtually non‐existent in Echimyidae (Bezerra & Oliveira, 2010; Pessôa & dos Reis, 1991; Raidan et al., 2019). Because some specimens had damaged axis, likely caused by collection methods, the final dataset comprised 99 atlas and 76 axis vertebrae. Specimens are housed in three Brazilian and two Argentinian institutions: Museu Nacional da Universidade Federal do Rio de Janeiro (Rio de Janeiro; MN), Museu de Zoologia da Universidade de São Paulo (São Paulo; MZUSP), Museu Paraense Emílio Goeldi (Belém; MPEG), Museo Municipal de Ciencias Naturales “Lorenzo Scaglia” (Mar del Plata; MMP) and Museo de La Plata (La Plata; MLP‐Mz). The list of all specimens used in this study is on Supplementary Table S1.

**FIGURE 2:**
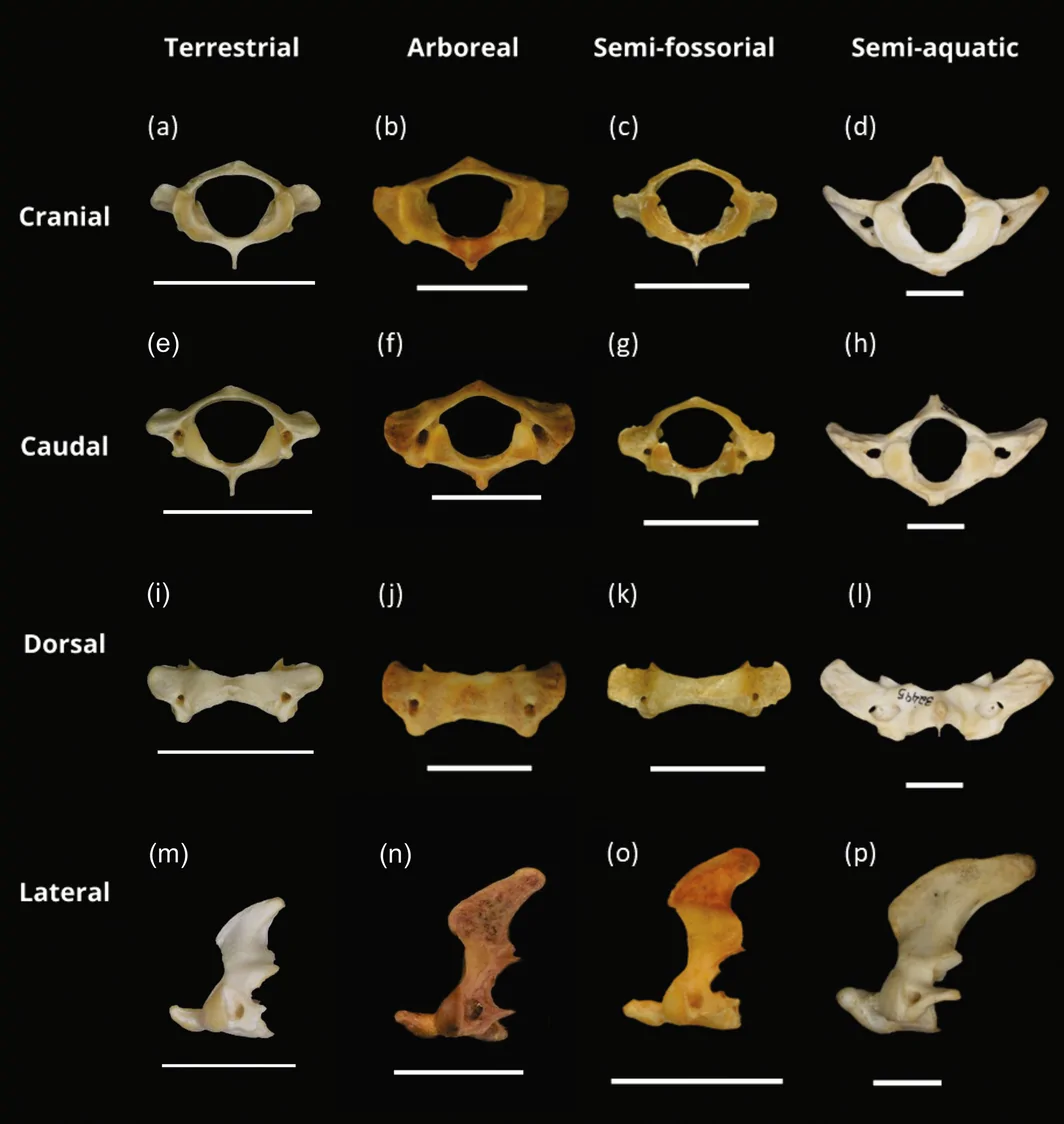
Examples of atlas and axis of Echimyidae in different views ordered according to locomotor modes. *Proechimys steerei* MPEG 28462(a, e, i, m); *Kannabateomys amblyonyx* (b, f, j, n), MN 81356 (b, f, j), MN 1961 (n); *Euryzygomatomys spinosus* MN 70164 (c, g, k, o); *Myocastor coypus* MZUSP 32495 (d, h, l, p). Scale bar 1 mm.

Each species was classified a priori by locomotor mode (terrestrial, arboreal, semi‐fossorial and semi‐aquatic; Figures 1 and 2), following previous studies (Fabre et al., 2013; Galewski et al., 2005; Netto & Tavares, 2021).

The atlas of each individual was photographed in three different views: cranial, caudal, and dorsal, while the axis was photographed in lateral view only (Figure 2). All photographs were taken using a Nikon D5300 digital camera with a Nikon Micro 40 mm *f*/2.8G lens at a fixed focal length. For each view, specimens were positioned on the same plane and at the same distance from the camera.

The morphological variation was described quantitatively using two‐dimensional (2D) geometric morphometrics. Although three‐dimensional methods are increasingly applied to complex structures, we used a 2D approach because it is less costly and satisfactorily captures the shape variation of the atlas and axis vertebrae. This approach requires photographs from multiple views, each of which was analyzed separately. Although the ventral view of the atlas vertebra could provide access to features such as the outline of the transverse process and the cranio‐caudal length of the vertebral arch, these aspects are already captured in the dorsal view. We analyzed the axis vertebra in lateral view only. Since the axis remains articulated with posterior vertebrae in most museum specimens, this configuration limits standardized cranial photography and makes caudal views virtually inaccessible without disarticulation. Moreover, its dorso‐ventrally elongated shape compromises coplanarity in dorsal view, making it unsuitable for 2D geometric morphometrics.

### 2.2 Geometric morphometrics

**FIGURE 3:**
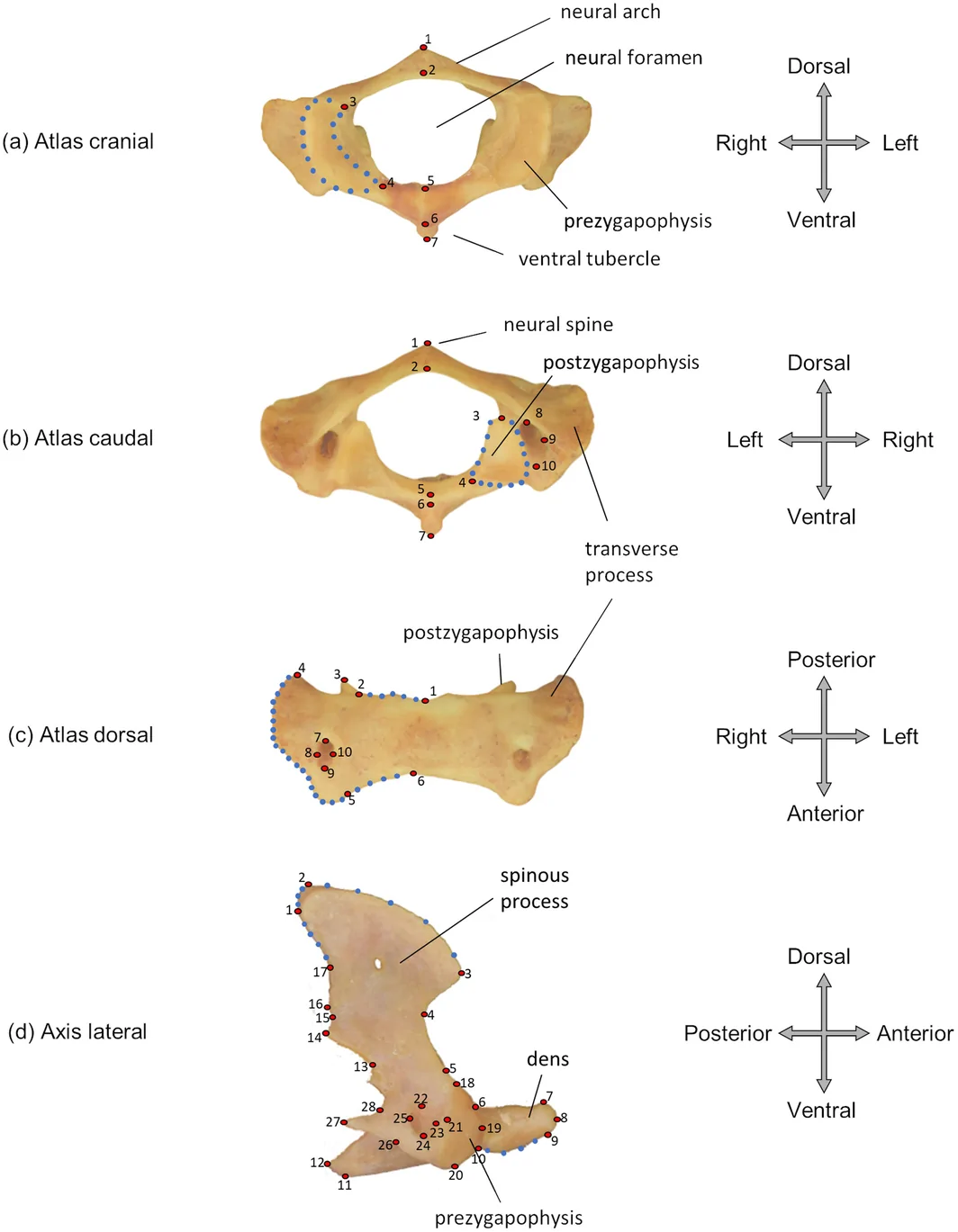
Landmark and semilandmark coordinate scheme for atlas and axis. Red circles, landmarks; blue circles, semilandmarks (descriptions in Supplementary Table S2).

We selected landmarks and semilandmarks based on prior studies to accurately represent vertebral morphology (Arlegi et al., 2022; Manfreda et al., 2006; Vander Linden, Campbell, et al., 2019; Vander Linden, Hedrick, et al., 2019; Villamil & Santiago‐Nazario, 2022). For each atlas, we digitized 7 landmarks and 20 semilandmarks in cranial view; 10 landmarks and 16 semilandmarks in caudal view; and 10 landmarks and 28 semilandmarks in dorsal view (Figure 3; Supplementary Table S2). For each axis, 28 landmarks and 16 semilandmarks were digitized in lateral view (Figure 3; Supplementary Table S2). The selected landmarks and semilandmarks were chosen to provide adequate coverage of morphology and satisfy the following criteria: homology, repeatability, consistency in relative position and coplanarity, and were based on previous studies with the same structures in other groups of mammals (Martín‐Serra et al., 2021; Monteiro & dos Reis, 1999; Vander Linden, Campbell, et al., 2019; Vander Linden, Hedrick, et al., 2019; Zelditch et al., 2012). Landmarks and semilandmarks were digitized on the right side of each specimen. When the right side was damaged, landmarks were digitized from the left side and mirrored. This approach is justified by the bilateral symmetry of vertebrae, which ensures anatomical consistency and minimizes potential error introduced by mirroring.

The *x* and *y* coordinates of landmarks and semilandmarks were digitized using the TpsDig 2.32 software (Rohlf, 2010a, 2015), the slider and link files were built using TpsUtil 1.81 (Rohlf, 2015) and the centroid sizes were computed with TPSRelw 1.74 (Rohlf, 2010b, 2015). We used Procrustes analysis in MorphoJ 1.07a (Klingenberg, 2011; Rohlf & Slice, 1990) to remove scale, orientation, and position effects. The resulting shape variables were then used in subsequent analyses. To minimize the errors associated with the digitization of landmarks and semilandmarks, each specimen and view were digitized twice by the same author (TFSN) and Procrustes analysis of variance (ANOVA) was applied using MorphoJ to assess the level of error produced. These analyses did not detect significant differences in size and shape between the replicates (Supplementary Table S3). Afterwards, we calculated the average for each species, which was used in subsequent analyses.

### 2.3 Phylogenetic comparative methods

For all phylogenetic comparative analyses, we used a time‐calibrated tree modified from Álvarez et al. (2017: p. 910; Figure 1). Although the echimyid phylogeny proposed by Courcelle et al. (2019) is more robust, the phylogenetic structure provided by Álvarez et al. (2017) comprises a broader taxonomic sampling, increasing the statistical power of phylogenetic comparative analyses (Paradis, 2014). The main differences between the topologies recovered in these two studies refer to the relationships among genera within Myocastorini. Nevertheless, we repeated our analyses using both phylogenies and the results found were similar; therefore, we chose to only show the results obtained using the phylogeny by Álvarez et al. (2017). The positioning of *E. chapalmalensis* on the phylogeny was based on Olivares et al. (2020). The phylogenetic topologies were manually edited, including the placement of *E. chapalmalensis*, using the TreeGraph 2 software (Stöver & Müller, 2010).

To test whether the morphological variation of the echimyid atlas and axis reflects their phylogenetic relatedness, the phylogenetic signal in the atlas and axis shapes was estimated using the generalized K statistic for multivariate data (*K*_mult_; Adams, 2014). *K*_mult_ values close to 1.0 suggest that closely related taxa share morphology consistent with Brownian Motion (BM) evolution. In contrast, *K*_mult_ ≪ 1.0 indicates less similarity among relatives than expected under BM, while *K*_mult_ ≫ 1.0 implies greater similarity (Blomberg et al., 2003). We estimated *K*_mult_ using the *physignal* function (geomorph 4.0.10 for R) with 1000 permutations.

Considering that morphometric data of related species are potentially interdependent due to shared ancestry (Felsenstein, 1985), we tested the evolutionary association of vertebral shape (shape variables) with vertebral size (log centroid size; CS), locomotor mode (LM), and their interaction (CS:LM) using Procrustes ANOVA within a phylogenetic generalized least squares (PGLS) framework. All analyses were performed with the function procD.pgls (geomorph 4.0.10; Baken et al., 2021). To identify the main axis of morphometric variation among echimyid species, we performed a principal component analysis (PCA) on shape variables using the *gm.prcomp* function of the geomorph 4.0.10 package for R (Baken et al., 2021). A preliminary inspection of the PCA results indicated that most of the variation between subfamilies, tribes, and locomotor modes is concentrated in the first two axes (PC1 and PC2), which together explain 50% or more of the total variation across all analyzed views. Subsequent axes showed substantial overlap among supraspecific taxa and little apparent phylogenetic and ecological structuring. Therefore, we focused on PC1 and PC2 for characterizing morphological variation among groups.

Given the large number of landmarks and the resulting high dimensionality of the data, we replicated the PGLS analyses using reduced datasets. First, we repeated the analyses using the leading PCs that together accounted for ≥90% of total shape variance (PC1–PC7 for all three atlas views; PC1–PC8 for the axis). Although these subsets and their corresponding PC scores do not fully represent overall shape variation, they capture the dominant biological signal while filtering out minor components that likely reflect residual variation or noise. This method has been similarly employed in prior studies to enhance interpretability while minimizing information loss (Jolliffe & Cadima, 2016; Lewton et al., 2020). Additionally, we conducted analyses using PC1 and PC2 both jointly and separately, in order to assess the dominant axes of shape variation and their individual contributions to evolutionary interpretations. Statistical significance was set at *α* = 0.05 for all analyses.

### 2.4 Evolutionary models

To investigate whether atlas and axis morphological evolution in echimyid lineages follows a single evolutionary regime or shows distinct adaptive peaks tailored to various locomotor modes, we evaluated 17 evolutionary models of continuous traits (Table 1). These models were fitted to PC scores (PC1 and PC2 for each view) from extant taxa, with model fit assessed using the small‐sample‐size‐corrected Akaike Information Criterion (AICc) in the R packages geiger (Harmon et al., 2008) and OUwie (Beaulieu et al., 2012). We first tested three base models assuming homogeneous evolution across the echimyid phylogeny: (1) a Brownian Motion model (BM1), representing random‐walk divergence under a single rate (*σ* ^2^); (2) an Ornstein–Uhlenbeck model (OU1), which adds stabilizing selection toward a fixed optimum (θ) with a constant strength (α); and (3) an Early Burst model (EB), which assumes a single evolutionary regime across the entire tree, regardless of clade or locomotor mode, but allows the evolutionary rate (σ^2^) to vary through time, typically decreasing exponentially as expected under early adaptive radiations (Beaulieu et al., 2012; Cadotte & Davies, 2016).

**TABLE 1:** Evolutionary models used in the study.

No. of optima	Model name	Description
0	Model 1: BM1	Random‐walk divergence under a single rate (*σ* ^2^)
1	Model 2: OU1	All locomotor modes sharing a single adaptive optimum (*θ*_Ter_ = *θ*_Aqu_ = *θ*_Fos_ = *θ*_Arb_)
0	Model 3: EB	Evolutionary rate (*σ* ^2^) exponentially decays over time
2	Model 4: OUM_Ter	Terrestrial species with a distinct adaptive optimum; other groups sharing the same optimum (*θ*_Ter_ ≠ *θ*_Aqu_ = *θ*_Fos_ = *θ*_Arb_)
2	Model 5: OUM_Aqu	Semi‐aquatic species with a distinct adaptive optimum; other groups sharing the same optimum (*θ*_Aqu_ ≠ *θ*_Ter_ = *θ*_Fos_ = *θ*_Arb_)
2	Model 6: OUM_Fos	Semi‐fossorial species with a distinct adaptive optimum; other groups sharing the same optimum (*θ*_Fos_ ≠ *θ*_Ter_ = *θ*_Aqu_ = *θ*_Arb_)
2	Model 7: OUM_Arb	Arboreal species with a distinct adaptive optimum; other groups sharing the same optimum (*θ*_Arb_ ≠ *θ*_Ter_ = *θ*_Aqu_ = *θ*_Fos_)
2	Model 8: OUM_Ter.Aqu_vs_Fos.Arb	Terrestrial and semi‐aquatic species sharing one optimum; semi‐fossorial and arboreal species sharing another (*θ*_Ter_ = *θ*_Aqu_ ≠ *θ*_Fos_ = *θ*_Arb_)
2	Model 9: OUM_Ter.Fos_vs_Aqu.Arb	Terrestrial and semi‐fossorial species sharing one optimum; semi‐aquatic and arboreal species sharing another (*θ*_Ter_ = *θ*_Fos_ ≠ *θ*_Aqu_ = *θ*_Arb_)
2	Model 10: OUM_Ter.Arb_vs_Aqu.Fos	Terrestrial and arboreal species sharing one optimum; semi‐aquatic and semi‐fossorial species sharing another (*θ*_Ter_ = *θ*_Arb_ ≠ *θ*_Aqu_ = *θ*_Fos_)
3	Model 11: OUM_Ter_vs_Aqu_vs_Fos.Arb	Terrestrial, semi‐aquatic, and the semi‐fossorial‐arboreal pair with distinct optima (*θ*_Ter_ ≠ *θ*_Aqu_ ≠ *θ*_Fos_ = *θ*_Arb_)
3	Model 12: OUM_Ter_vs_Fos_vs_Aqu.Arb	Terrestrial, semi‐fossorial, and the semi‐aquatic‐arboreal pair with distinct optima (*θ*_Ter_ ≠ *θ*_Fos_ ≠ *θ*_Aqu_ = *θ*_Arb_)
3	Model 13: OUM_Ter_vs_Arb_vs_Aqu.Fos	Terrestrial, arboreal, and the semi‐aquatic‐semi‐fossorial pair with distinct optima (*θ*_Ter_ ≠ *θ*_Arb_ ≠ *θ*_Aqu_ = *θ*_Fos_)
3	Model 14: OUM_Aqu_vs_Fos_vs_Ter.Arb	Semi‐aquatic, semi‐fossorial, and the terrestrial‐arboreal pair with distinct optima (*θ*_Aqu_ ≠ *θ*_Fos_ ≠ *θ*_Ter_ = *θ*_Arb_)
3	Model 15: OUM_Aqu_vs_Arb_vs_Ter.Fos	Semi‐aquatic, arboreal, and the terrestrial‐semi‐fossorial pair with distinct optima (*θ*_Aqu_ ≠ *θ*_Arb_ ≠ *θ*_Ter_ = *θ*_Fos_)
3	Model 16: OUM_Fos_vs_Arb_vs_Ter.Aqu	Semi‐fossorial, arboreal, and the terrestrial‐semi‐aquatic pair with distinct optima (*θ*_Fos_ ≠ *θ*_Arb_ ≠ *θ*_Ter_ = *θ*_Aqu_)
4	Model 17: OUM_all	Each locomotor mode with its own adaptive optimum (*θ*_Ter_ ≠ *θ*_Aqu_ ≠ *θ*_Fos_ ≠ *θ*_Arb_)

Abbreviations: Arb, arboreal; Aqu, semi‐aquatic; BM1, Brownian Motion; EB, Early Burst; Fos, semi‐fossorial; OU1, Ornstein–Uhlenbeck with single optimum; OUM, Ornstein–Uhlenbeck with multiple optima; Ter, terrestrial.

We then evaluated more complex Ornstein–Uhlenbeck models (OUM) with multiple adaptive peaks, which allow the optimal trait value (*θ*) to vary across phylogenetic branches according to locomotor mode (Table 1). These models describe trait evolution under distinct selective regimes, each associated with different locomotor modes, where phenotypes are attracted toward their respective adaptive optima (*θ*) (Beaulieu et al., 2012; Cadotte & Davies, 2016). The simplest and least parameterized OUM model variants were those in which only one locomotor mode was assigned a distinct adaptive optimum, while the remaining modes shared a common regime (OUM_Ter, OUM_Aqu, OUM_Fos, and OUM_Arb; Models 4–7 in Table 1). The most complex and parameter‐rich model in our analysis was OUM_all (Model 17 in Table 1), assigning a distinct adaptive optimum to each locomotor mode. We also evaluated a set of intermediate models that assigned different optima to specific groupings of locomotor modes (Models 8–16 in Table 1). These models tested more nuanced hypotheses of ecological similarity among subsets of locomotor modes. A complete description of all OUM variants is provided in Table 1. The likelihood calculations for these models, particularly the multi‐parameter OUM, were contingent on an accurate mapping of locomotor modes within the phylogenetic framework. We executed 1000 stochastic mappings of these traits using the *make.simmap* function from the phytools package to account for random variation influencing model likelihood and AICc values. Then, the modal values of likelihood and AICc for each model were calculated. We selected the model with the lowest corrected AICc value. All models with ΔAICc ≤3.5 were deemed to have equivalent suitability (Burnham & Anderson, 2002).

Although previous studies have shown that fitting evolutionary models to individual principal components (PCs) can introduce bias, particularly by favoring EB models (Adams & Collyer, 2018; Clavel et al., 2015; Uyeda et al., 2015), our analyses revealed that the EB model did not provide a good fit for any of the PCs in our dataset (see Results). Rather than testing all components, we sought to investigate which evolutionary models best explain the primary axes of morphological variation, namely those in which clearer phylogenetic or functional patterns were visually identifiable. We therefore focused on PC1 and PC2 from each anatomical view, as these components captured the largest proportions of shape variation and were more biologically interpretable. We chose not to use multivariate evolutionary models, such as those implemented in the mvMORPH package (Clavel et al., 2015), due to the high dimensionality of our data relative to the number of taxa, which could result in overparameterization and unstable estimates. Moreover, mvMORPH does not allow for variation in selective optima or evolutionary rates across regimes under the OU model, limiting its applicability for testing functional hypotheses.

### 2.5 Inference of locomotor modes in Echimyidae

To infer the locomotor strategy employed by the extinct *E. chapalmalensis*, we used the shape data from the extant taxa with known locomotor modes to “train” a linear discriminant analysis (LDA). The resulting discriminant functions from this training set were then used to classify *E. chapalmalensis* into one of the sampled locomotor modes. To compensate for the different sample sizes for each locomotor mode, we used the same prior probability for all modes in the training set. We were unable to include semi‐aquatic locomotion in these analyses, as there was only one species with this mode in our sample.

Using shape variables directly in LDA is problematic, as group sample sizes are usually smaller than the number of variables. In LDA, increasing the number of variables relative to the number of specimens/species (*N*) will result in better group classification. However, if the number of variables is equal to or exceeds N, then discriminant functions may separate groups even if all specimens come from the same population and no group structure actually exists (Mitteroecker & Bookstein, 2011). This happens because the number of dimensions in the morphospace also increases with the number of variables, which in turn increases the number of ways data can be rotated. With enough dimensions, any group separation can be achieved (Bookstein, 2002), though devoid of any biological meaning. Given that our sample includes only three semi‐fossorial species, we retained only the first two PCs for the LDAs. The LDAs were performed while accounting for phylogenetic signal in the data. To minimize potential biases from sampling limitations, we also conducted 1000 bootstrap iterations. In each iteration, specimens/species within locomotor groups were randomly resampled to “train” new LDAs, after which *E. chapalmalensis* was reclassified using the new discriminant functions. The LDAs were performed using the MASS package for R (Venables & Ripley, 2002).

## 3 RESULTS

### 3.1 Phylogenetic signal

When analyzing 100% of shape variation, *K*_mult_ estimates for the three atlas views and the lateral axis view ranged from 0.587 to 0.795 (*p* <0.001; Table 2), indicating moderate phylogenetic signal. The strongest signal was found in the caudal view of the atlas. These results highlight the importance of accounting for the phylogenetic structure of phenotypic variation and covariation in the subsequent comparative analyses.

**TABLE 2:** Multivariate phylogenetic signal (*K*_mult_) assessed on Procrustes coordinates for the atlas and axis of echimyids.

View	*K*_mult_	*p*
Atlas cranial	0.743	0.001
Atlas caudal	0.795	0.001
Atlas dorsal	0.587	0.001
Axis lateral	0.616	0.001

### 3.2 Allometry

When the full datasets (all shape variation) were analyzed, PGLS detected no significant correlation between vertebral size and shape in the cranial view of the atlas (*R* ^2^ = 0.086, *p* = .086), but significant correlations in the caudal and dorsal views of the atlas and in the lateral view of the axis (0.106 ≤*R* ^2^ ≤ 0.211; *p* ≤.020; Table 3). The same general pattern was recovered when analyzing the reduced dataset retaining 90% of total shape variation (Table 3). When dimensionality was further reduced, PGLS analyses of PC1 and PC2 combined revealed significant correlations between centroid size and shape in all views, with allometric effects being more pronounced in the dorsal view of the atlas and the lateral view of the axis (*R* ^2^ ≥0.407; *p* <0.001; Table 3). When PC1 was analyzed separately, the allometric effect was significant in the cranial and dorsal views of the atlas and in the lateral view of the axis, but not in the caudal view of the atlas (Table 3). In contrast, PC2 showed significant allometric effects in the caudal and dorsal views of the atlas and in the lateral view of the axis, but not in the cranial view of the atlas (Table 3). Shape changes in the atlas associated with increasing size included the relative enlargement of the joint surfaces of the posterior apophyses, dorso‐ventral elongation of the neural spine, dorso‐ventral shortening of the ventral tubercle, and lateral expansion of the transverse process. In the axis, size increase was associated with cranio‐caudal and dorso‐ventral expansion of the neural spine.

**TABLE 3:** Phylogenetic regression analyses (PGLS) among shape variables (Total), PCs representing 90% of the total shape variation, and the first two Principal components with size, locomotor mode, and the interaction between size and locomotor mode as variables. The size is defined by Log_10_ centroid size (CS), and the locomotor mode (LM) is classified into four categories (terrestrial, arboreal, semi‐fossorial and semi‐aquatic; see Figure 1).

		PGLS					
		*R* ^2^	*F*	*p*	*R* ^2^	*F*	*p*
		Atlas cranial			Atlas caudal		
Total	CS	0.086	1.937	0.086	0.106	2.657	0.020*
	LM	0.229	1.289	0.204	0.222	1.393	0.111
	CS:LM	0.061	0.686	0.659	0.073	0.919	0.516
90%	CS	0.094	2.170	0.059	0.117	2.972	0.022*
	LM	0.250	1.438	0.140	0.231	1.467	0.115
	CS:LM	0.046	0.526	0.809	0.062	0.783	0.609
PC1 + PC2	CS	0.147	5.247	0.013*	0.151	4.636	0.022*
	LM	0.408	3.630	0.008**	0.311	2.393	0.039*
	CS:LM	0.052	0.927	0.443	0.049	0.762	0.493
PC1	CS	0.190	7.451	0.024*	0.011	0.497	0.489
	LM	0.409	4.004	0.024*	0.540	5.891	0.003**
	CS:LM	0.044	0.865	0.424	0.104	2.270	0.136
PC2	CS	0.077	2.390	0.162	0.240	6.200	0.025*
	LM	0.406	3.146	0.058	0.165	1.071	0.409
	CS:LM	0.065	1.008	0.388	0.015	0.192	0.755
		Atlas dorsal			Axis lateral		
Total	CS	0.211	6.112	<0.001***	0.183	4.783	<0.001***
	LM	0.218	1.574	0.076	0.226	1.477	0.070
	CS:LM	0.087	1.253	0.282	0.093	1.220	0.267
90%	CS	0.247	7.668	<0.001***	0.209	5.881	<0.001***
	LM	0.229	1.779	0.049*	0.247	1.740	0.032*
	CS:LM	0.073	1.134	0.356	0.081	1.142	0.329
PC1 + PC2	CS	0.428	23.259	<0.001***	0.407	15.22	<0.001***
	LM	0.229	3.115	0.034*	0.157	1.465	0.240
	CS:LM	0.085	2.305	0.119	0.089	1.657	0.204
PC1	CS	0.501	29.639	<0.001***	0.547	18.016	0.002**
	LM	0.190	2.805	0.085	0.039	0.321	0.850
	CS:LM	0.072	2.132	0.183	0.019	0.308	0.751
PC2	CS	0.216	9.486	0.015*	0.198	9.279	0.021*
	LM	0.344	3.783	0.029*	0.332	3.902	0.023*
	CS:LM	0.122	2.678	0.096	0.193	4.531	0.023*

*Note*: *F*, *F*‐statistics; *R* ^2^, regression coefficient; *p*, *p*‐value; *p <0.05; **p <0.01; ***p <0.001.

### 3.3 Locomotor modes

PGLS analysis of all shape variables revealed no significant association between vertebral shape and locomotor mode in any of the views examined (0.217 <*R* ^*2*^ < 0.229; *p* ≥0.070). However, when dimensionality was reduced by retaining PCs that accounted for 90% of shape variation, PGLS revealed significant associations between locomotor mode and shape in the dorsal view of the atlas and in the lateral view of the axis (0.229 ≤*R* ^2^ ≤0.247; *p* ≤0.049), but not in the cranial or caudal views of the atlas (*p* ≥0.115; Table 3). When dimensionality was further reduced to include only PC1 and PC2, PGLS analyses revealed significant associations between locomotor mode and shape in the cranial, caudal, and dorsal views of the atlas (0.229 ≤*R* ^2^ ≤0.407; *p* ≤0.039), but not in the lateral view of the axis (*R* ^2^ = 0.156; *p* = 0.240; Table 3). When PCs were analyzed separately, PGLS revealed that for PC1, locomotor mode was significantly associated with shape in the cranial (*R* ^2^ = 0.408, *p* = 0.024) and caudal (*R* ^2^ = 0.540, *p* = 0.003) views of the atlas, but not in the dorsal view of the atlas or the lateral view of the axis (*p* ≥0.085). For PC2, significant associations were found in the dorsal view of the atlas (*R* ^2^ = 0.344, *p* = 0.029) and in the lateral view of the axis (*R* ^2^ = 0.332, *p* = 0.023), whereas no significant effects were detected in the cranial or caudal views of the atlas (*p* ≥0.058; Table 3).

Across all views and data subsets, a significant association between vertebral shape and the interaction between centroid size and locomotor mode (CS:LM) was detected only for PC2 of the lateral view of the axis. No significant effects were found in any other case (Table 3).

**FIGURE 4:**
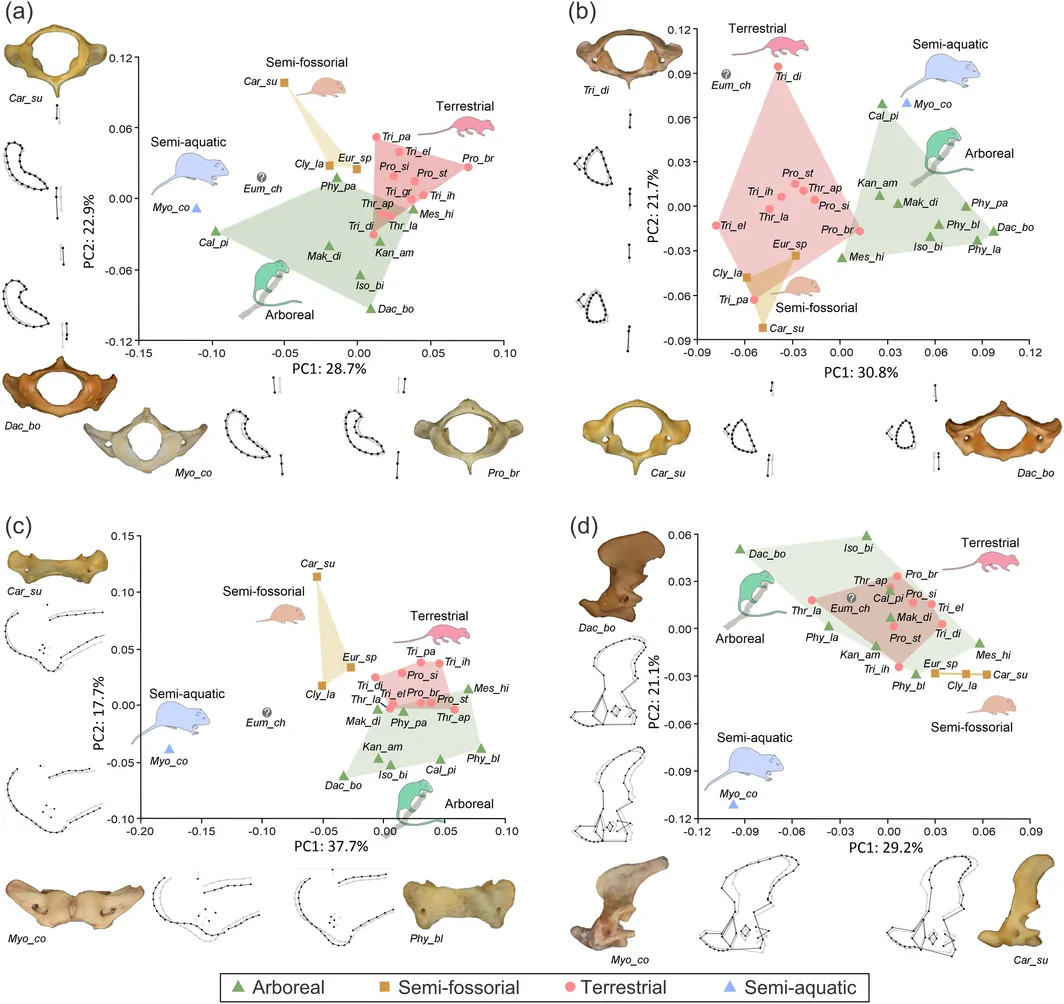
Principal component analysis (PC1 vs. PC2) of the echimyid atlas and axis vertebrae. (a)–(c), atlas, (a), cranial view; (b), caudal view; (c), dorsal view; (d), axis in lateral view. The convex hull represents the distribution of each locomotor mode in the morphospace. Black wireframes show the change in shape while light gray wireframes show the average shape. Abbreviations for the Echimyidae species are in Figure 1.

For the atlas shape, species with different locomotor modes tended to occupy distinct regions along the morphospace formed by PC1 and PC2 (Figure 4a–c), while showing substantial overlap along PC3 and subsequent axes. In the atlas, differentiation was greatest in the caudal view (Figure 4b), followed by the dorsal and cranial views (Figure 4a,c). In the lateral view of the axis, there was more overlap among locomotor modes, with only subtle differentiation along PC2 (Figure 4d). The EB model was never the best‐fitting model in any of the analyses (all ΔAICc >3.5; Tables 1 and 4). In contrast, multi‐regime models positing distinct adaptive optima for at least one locomotor mode (e.g., OUM) were among the best‐fitting models for PC1 and PC2 (Tables 1 and 4). Below, we describe the shape variation based on PCA for each view analyzed and present the evolutionary models that best explain this variation.

**TABLE 4:** Relative fit of evolutionary models to PC1 and PC2 for each view of echimyid atlas (A–C) and axis (D).

		A. Cranial view of atlas						B. Caudal view of atlas					
		PC1_cranial_			PC2_cranial_			PC1_caudal_			PC2_caudal_		
Model	*k*	Log.Lik	AICc	ΔAICc	Log.Lik	AICc	ΔAICc	Log.Lik	AICc	ΔAICc	Log.Lik	AICc	ΔAICc
1	2	38.543	−72.419	4.940	42.375	−80.084	**0.145**	40.982	−77.298	9.906	36.558	−68.450	**2.706**
2	3	38.543	−69.674	7.685	42.375	−77.338	**2.890**	40.982	−74.553	12.651	37.934	−68.456	**2.699**
3	3	38.578	−69.746	7.614	43.369	−79.328	**0.900**	41.207	−75.003	12.201	36.558	−65.705	5.451
4	4	39.699	−68.898	8.462	42.456	−74.411	5.817	42.731	−74.963	12.241	38.211	−65.922	5.234
5	4	41.928	−73.355	4.004	42.383	−74.266	5.962	41.459	−72.419	14.785	39.639	−68.779	**2.377**
6	4	38.850	−67.200	10.160	44.327	−78.154	**2.074**	41.844	−73.188	14.016	40.702	−70.904	**0.251**
7	4	38.752	−67.003	10.356	44.395	−78.291	**1.937**	45.306	−80.111	7.093	37.966	−65.433	5.723
8	4	39.529	−68.558	8.801	42.624	−74.747	5.481	42.112	−73.724	13.480	39.242	−67.983	**3.172**
9	4	39.403	−68.305	9.054	45.364	−80.228	0.000*	48.852	−87.204	0.000*	38.319	−66.138	5.018
10	4	40.466	−70.432	6.928	43.922	−77.343	**2.885**	41.327	−72.154	15.050	38.710	−66.920	4.235
11	5	45.680	−77.360	0.000*	42.650	−71.299	8.929	42.874	−71.748	15.456	40.553	−67.107	4.049
12	5	41.699	−69.399	7.961	46.142	−78.284	**1.944**	49.091	−84.181	**3.023**	40.840	−67.679	**3.476**
13	5	41.944	−69.888	7.472	43.922	−77.343	**2.885**	48.821	−83.641	3.563	39.762	−65.524	5.632
14	5	42.794	−71.589	5.771	44.467	−74.934	5.294	42.233	−70.466	16.738	42.578	−71.156	0.000*
15	5	42.537	−71.074	6.285	45.333	−76.667	3.561	48.826	−83.652	3.552	39.736	−65.472	5.683
16	5	39.145	−64.291	13.069	46.558	−79.117	**1.112**	45.609	−77.217	9.987	41.204	−68.407	**2.748**
17	6	38.828	−67.157	6.679	46.626	−76.275	3.953	41.850	−73.201	6.929	42.808	−67.617	3.539

Model	*k*	C. Dorsal view of atlas						D. Lateral view of axis					
		PC1_dorsal_			PC2_dorsal_			PC1_lateral_			PC2_lateral_		
		Log.Lik	AICc	ΔAICc	Log.Lik	AICc	ΔAICc	Log.Lik	AICc	ΔAICc	Log.Lik	AICc	ΔAICc
1	2	31.704	−58.741	19.675	39.796	−74.924	6.020	31.704	−58.741	19.615	38.798	−72.890	11.327
2	3	32.284	−57.157	21.259	39.903	−72.394	8.550	32.284	−57.157	21.198	39.131	−70.762	13.456
3	3	31.703	−55.996	22.420	39.795	−72.179	8.765	34.762	−62.025	16.331	38.798	−70.096	14.122
4	4	33.174	−55.848	22.568	40.282	−70.064	10.881	33.121	−55.742	22.614	39.708	−68.749	15.468
5	4	40.290	−70.080	8.336	40.261	−70.022	10.922	40.299	−70.097	8.258	46.054	−81.441	**2.777**
6	4	33.267	−56.034	22.382	42.289	−74.077	6.867	33.213	−55.927	22.429	39.895	−69.124	15.094
7	4	33.054	−55.608	22.808	42.642	−74.785	6.160	33.056	−55.611	22.745	40.016	−69.365	14.852
8	4	32.429	−54.359	24.057	40.159	−69.818	11.126	32.412	−54.324	24.032	39.336	−68.005	16.213
9	4	32.415	−54.331	24.085	45.262	−80.023	**0.921**	32.381	−54.263	24.093	39.209	−67.752	16.466
10	4	39.707	−68.915	9.501	41.134	−71.768	9.176	39.659	−68.817	9.538	44.606	−78.546	5.672
11	5	41.031	−68.061	10.354	40.476	−66.952	13.992	40.990	−67.981	10.375	45.954	−77.622	6.596
12	5	33.787	−53.573	24.843	47.472	−80.944	0.000*	33.787	−53.573	24.782	40.187	−66.088	18.130
13	5	39.672	−65.343	13.073	43.800	−73.601	7.344	39.805	−65.610	12.745	46.373	−78.460	5.757
14	5	46.208	−78.416	0.000*	42.698	−71.395	9.549	46.178	−78.356	0.000*	49.252	−84.218	0.000*
15	5	40.584	−67.169	11.247	45.041	−76.083	4.861	40.600	−67.200	11.156	46.310	−78.334	5.883
16	5	33.641	−53.283	25.133	45.631	−77.261	3.683	33.573	−53.146	25.210	40.411	−66.536	17.681
17	6	46.246	−74.493	3.923	47.580	−77.106	3.838	46.223	−74.282	4.074	49.273	−80.084	4.134

*Note*: *k*, number of parameters in the model; log.lik, log‐likelihood of models; AICc, Akaike information criterion values corrected for small sample sizes; ΔAICc, AICc difference relative to the lowest AICc value; *, models with the lowest AICc (ΔAICc = 0.0); in bold, ΔAICc <3.5. Details of the evolutionary models are presented in Table 1.

*Cranial view of atlas*. PC1_cranial_ (28.7% of shape variation) primarily describes variation in the shape of the prezygapophyses (Figure 4a). Along this axis, terrestrial species exhibit high scores, with extreme phenotypes observed in *Proechimys brevicauda* and *Trinomys iheringi* (Figure 4a). This terrestrial configuration is characterized by prezygapophyses with a pronounced concavity along the medial margin, a medio‐laterally compressed dorsal portion, and an expanded ventral portion. At the opposite end of the axis, the semi‐aquatic *Myocastor coypus*, the arboreal *Callistomys pictus*, the fossil *E. chapalmalensis*, and the semi‐fossorial *Carterodon sulcidens* share very low PC1_cranial_ scores, which reflect prezygapophyses lacking a marked medial concavity. The arboreal species *Mesomys hispidus*, *Kannabateomys amblyonyx*, and *Dactylomys boliviensis* overlap with terrestrial species along this axis, presenting relatively high scores. The remaining arboreal and semi‐fossorial species display intermediate scores and correspondingly intermediate morphologies (Figure 4a).

The only evolutionary model supported for PC1_cranial_ was Model 11 (ΔAICc ≤3.5; Tables 1 and 4), suggesting that terrestrial and semi‐aquatic locomotor modes each have distinct adaptive optima, whereas semi‐fossorial and arboreal modes converge toward a shared optimum.

PC2_cranial_ explained 22.9% of the shape variation in the cranial view and mainly captures variation in the relative length of the ventral tubercle and the dorso‐ventral height of the neural foramen. The arboreal *D. boliviensis* showed the lowest score, followed by most other arboreal species, indicating that they share a short ventral tubercle and a relatively low neural foramen. At the opposite extreme, semi‐fossorial species, particularly *C. sulcidens*, and some terrestrial species, most notably *Trinomys paratus* and *Trinomys eliasi*, exhibited high scores, reflecting a long ventral tubercle and a relatively tall neural foramen. The semi‐aquatic *M. coypus*, most other terrestrial species, as well as the extinct *E. chapalmalensis*, showed intermediate scores and corresponding intermediate morphologies (Figure 4a).

The best evolutionary model explaining variation along PC2_cranial_ was the multi‐optima Model 9 (Tables 1 and 4). However, other models, including the single‐regime model (BM1) and other multiple‐regime models, also showed similar suitability (ΔAICc ≤3.5; Table 4).

*Caudal view of atlas*. PC1_caudal_ (30.8% of shape variation) primarily describes the shape of the postzygapophyses, the length of the ventral tubercle, and the height of the neural spine. Along this axis, there is a differentiation between arboreal and semi‐aquatic species, which show high scores (with the highest in *D. boliviensis*), and terrestrial and semi‐fossorial species, as well as *E. chapalmalensis*, which exhibit low scores (with the lowest in *T. eliasi*). This indicates that arboreal and semi‐aquatic species tend to have rounded postzygapophyses, a long neural spine, and a reduced ventral tubercle. In contrast, terrestrial and semi‐fossorial species are characterized by triangular postzygapophyses, a short neural spine, and an expanded ventral tubercle. A slight overlap between groups is observed: the terrestrial *P. brevicauda* and the arboreal *M. hispidus* show intermediate scores near the center of the axis (Figure 4b).

The best model for PC1_caudal_ was Model 9, while Model 12 showed comparable support (ΔAICc = 3.023; Tables 1 and 4). These results indicate that terrestrial and semi‐fossorial locomotor modes share one adaptive optimum, whereas semi‐aquatic and arboreal modes converge on another.

Along PC2_caudal_ (21.7% of shape variation), there is substantial overlap among locomotor modes. Additionally, the best‐fitting evolutionary model for this axis was Model 14, although several alternative models, including the single‐regime models BM1 and OU1, also showed comparable support (ΔAICc <3.5; Tables 1 and 4).

*Dorsal view of atlas*. PC1_dorsal_ (37.7% of shape variation) primarily describes the lateral projection of the transverse process and the cranio‐caudal extension of the articular facets (postzygapophyses). Arboreal and terrestrial species largely overlap and exhibit high scores, with the highest values observed in the arboreal *Phyllomys blainvillii* and *M. hispidus*. At the opposite extreme, the semi‐aquatic *M. coypus*, followed by the extinct *E. chapalmalensis* shows distinctly low scores, setting them apart from all other species. Semi‐fossorial species occupy an intermediate position but are morphologically closer to arboreal and terrestrial species than to *M. coypus* (Figure 4c). This pattern indicates that arboreal and terrestrial species tend to have more projected postzygapophyses and laterally reduced transverse processes, while *M. coypus*—and to a lesser extent, *E. chapalmalensis*—exhibit less projected postzygapophyses and laterally expanded transverse processes.

Among all tested evolutionary models, the best‐fitting one explaining score variation along PC1_dorsal_ was Model 14, indicating that semi‐aquatic and semi‐fossorial locomotor modes have independent adaptive optima, whereas terrestrial and arboreal modes share a common adaptive optimum (Tables 1 and 4).

PC2_dorsal_ (17.7% of shape variation) shows greater overlap among locomotor modes than PC1_dorsal_, although a slight separation is observed between semi‐fossorial/terrestrial species—led by the semi‐fossorial *C. sulcidens* with the highest score—and arboreal/semi‐aquatic species, with the arboreal *D. boliviensis* exhibiting the lowest score. This axis mainly captures variation in the cranio‐caudal thickness of the neural arches. Semi‐fossorial species, especially *C. sulcidens*, and terrestrial species are characterized by cranio‐caudally narrowed neural arches. In contrast, semi‐aquatic and arboreal species exhibit cranio‐caudally expanded neural arches (Figure 4c).

The Model 12 best explained variation along PC2_dorsal_, although Model 9 showed similar support (ΔAICc = 0.921; Tables 1 and 4).

*Lateral view of the axis*. PC1_lateral_ (29.2% of shape variation) primarily describes the cranio‐caudal length of the spinous process (Figure 4d). The semi‐aquatic *M. coypus* shows the lowest PC1 score, indicating a distinctly elongated spinous process and standing apart from all other species. At the opposite end, the semi‐fossorial *C. sulcidens* exhibits the highest score, corresponding to a cranio‐caudally compressed spinous process. Other semi‐fossorial species also tend to score higher than most arboreal and terrestrial taxa, though with some degree of overlap. *E. chapalmalensis* shows intermediate PC1 values, overlapping with arboreal and terrestrial species. These two groups span a broad range of scores, with no clear separation between them.

The best‐fitting model explaining variation along PC1_lateral_ was Model 14 (Tables 1 and 4).

Along PC2_lateral_ (21.1% of shape variation), which primarily reflects variation in vertebral body length, *M. coypus* exhibits a markedly lower score than all other species, corresponding to an extremely cranio‐caudally compressed vertebral body and short dens—the most pronounced phenotype along this axis. Semi‐fossorial species also show lower scores than arboreal and terrestrial taxa, suggesting a tendency toward vertebral body compression, although their morphology remains much closer to the latter groups than to *M. coypus*. In contrast, arboreal and terrestrial species generally exhibit higher scores, reflecting a cranio‐caudally elongated vertebral body and long dens.

The best‐fitting model for PC2_lateral_ was Model 14, followed by Model 5 (ΔAICc <3.5; Tables 1 and 4).

### 3.4 Linear discriminant analysis

The LDAs satisfactorily confirmed morphological differentiation between locomotor modes among living species. The correct reclassification of locomotor modes in extant species ranged from 68.42% (axis) to 95.24% (caudal view of the atlas; Table 5). The LDAs of the atlas caudal view recovered terrestrial mode for *E. chapalmalensis* with posterior probabilities of 98.63% (Table 5), whereas the LDAs recovered the semi‐fossorial mode in the other views (posterior probabilities ranging from 66.5 to 99.9%). All these results indicate moderate to high posterior probabilities and were corroborated by most bootstrap iterations (Supplementary Tables S4 and S5).

**TABLE 5:** Linear discriminant analysis.

View	% reclassification				% posterior probability (fossil)		
	Terrestrial	Arboreal	Semi‐fossorial	Total	Terrestrial	Arboreal	Semi‐fossorial
Atlas							
Caudal	88.8	100	100	95.2	**98.6**	0.0	1.4
Cranial	80.0	71.4	100	80	8.4	25.1	**66.5**
Dorsal	77.8	75.0	100	80	0.1	0.0	**99.9**
Axis							
Lateral	62.5	62.5	100	68.4	**47.9**	41.6	10.5

*Note*: Percentage of echimyids “correctly” reclassified to their original locomotor mode and the inferred locomotor modes for the extinct *Eumysops chapalmalensis* with their respective posterior probabilities for the caudal, cranial and dorsal views of the atlas, and for the lateral view of the axis. Results obtained using linear discriminant analyses trained on the average species values dataset and taking into account the phylogenetic structure of the data.

The LDAs of the lateral view of the axis generally did not effectively discriminate between arboreal and terrestrial taxa, with a correct reclassification rate of only 62.5%. In these analyses, *E. chapalmalensis* was consistently classified as either terrestrial or arboreal, with semi‐fossorial being the least probable locomotion mode. Notably, in the LDAs that classified *E. chapalmalensis* as terrestrial, the posterior probabilities for arboreal and terrestrial locomotion were similar (~41% and ~48%, respectively; Table 5). These ambiguous results were corroborated by the bootstrap analyses, which showed only a slight increase in posterior probabilities (Supplementary Table S5).

## 4 DISCUSSION

The atlas‐axis complex represents a key anatomical innovation that allowed mammals to develop and specialize in ecologically significant behaviors (Evans, 1939). Compared to other tetrapods, mammals exhibit exceptionally low variability in the number of cervical vertebrae, with all species following the “rule of seven,” except for sloths and manatees (Buchholtz et al., 2014; Diogo et al., 2008; Galis, 1999; Narita & Kuratani, 2005; Terray et al., 2020). Our results indicate that the constraint imposed by this fixed number does not lead to low morphological diversity, at least not for the atlas and axis. The shape variation observed within Echimyidae suggests that the morphology of these vertebrae reflects phylogenetic history and, to a lesser extent, allometric and locomotor modes.

Morphological differences in cervical vertebrae among mammals can arise from both phylogenetic distance and distinct locomotor modes or lifestyles (Johnson et al., 1999). In echimyids, we detected moderate phylogenetic signal in the morphology of both the atlas and the axis (Table 2). These findings align with previous research across various mammalian groups—including Monotremata, Marsupialia, Laurasiatheria, Euarchontoglires (especially Primates), Afrotheria, and Xenarthra—which also reported moderate phylogenetic signal for atlas and axis shape (Vander Linden, Campbell, et al., 2019; Vander Linden, Hedrick, et al., 2019), and small‐ to medium‐sized mammals with an average weight of up to 5 kg (Taewcharoen et al., 2024). In our dataset, this moderate phylogenetic signal was associated with overlap among major clades in the morphospaces, which hinders the identification of unequivocal morphological markers for each lineage.

Initially, the high dimensionality inherent to morphometric datasets, especially those based on large numbers of landmarks and semilandmarks, may obscure potential evolutionary or ecological signals. Despite this, our PGLS analyses of the shape variables revealed significant relationships between vertebral shape and size across all examined views, except for the cranial view of the atlas. In contrast, no significant association with locomotor mode was detected in any view (Table 3). Thus, to increase the statistical power of subsequent analyses, we reduced dimensionality by focusing on the subset of PCs accounting for ≥90% of cumulative shape variance and by conducting additional analyses restricted to PC1 and PC2 (jointly and separately). Although this approach may incorporate non‐allometric variation that could influence results (Klingenberg, 2016), we deemed it methodologically justified to emphasize major axes of biological variation while minimizing residual components. This strategy aligns with previous work conducted in Carnivora (Lewton et al., 2020), where it effectively isolated biologically meaningful signals on cervical vertebrae. In our study, these reduced configurations consistently revealed phylogenetic signal, moderate allometric effects, and significant associations between vertebral shape and locomotor mode across all views analyzed (Tables 2 and 3).

### 4.1 Allometric effects

In mammals, increases in body size are commonly associated with two main morphological trends in the cervical vertebrae: (i) allometric changes in vertebral shape, and (ii) relative shortening of the neck to enhance head stability (Arnold et al., 2017; Vander Linden, Campbell, et al., 2019). Among echimyids, our findings suggest that while allometry significantly influences the shape of the atlas and axis, its overall effect is limited. Although allometric patterns were statistically significant, the proportion of shape variation explained by centroid size was generally low (*R* ^2^ < 0.21; Table 3). After dimensionality reduction, the explanatory power increased slightly in some cases but remained weak to moderate overall (*R* ^2^ < 0.25), with higher values observed when analyses were restricted to PC1 or PC1 + PC2 in the dorsal view of the atlas and the lateral view of the axis. These results indicate that allometric effects are anatomically localized and context‐dependent. For most views and configurations, locomotor mode accounted for more shape variation than vertebral size (Table 3). Overall, centroid size is not the predominant factor shaping vertebral morphology but acts in combination with other influences in their evolutionary diversification.

Nevertheless, the anatomical components most responsive to body size variation shed light on potential functional demands. In the atlas, increases in vertebral size were associated with enlargement of the articular surfaces of the anterior and posterior apophyses, dorso‐ventral elongation of the neural spine, shortening of the ventral tubercle, elongation of the neural arch, and lateral expansion of the transverse processes. In the axis, larger species exhibited cranio‐caudal and dorso‐ventral expansion of the spinous process. These morphological trends are biomechanically consistent with the need to support a heavier head and increased development of the cervical musculature, particularly the nuchal muscles involved in head stabilization and movement (Hatt, 1932; Vander Linden, Hedrick, et al., 2019).

Previous studies have also identified size–shape correlations in cervical vertebrae of pinnipeds, tupaiids, primates, and other rodents, even after accounting for phylogenetic structure (Pierce et al., 2011; Sargis, 2001; Vander Linden, Campbell, et al., 2019; Vander Linden, Hedrick, et al., 2019). Our findings are broadly consistent with these observations, though the strength of the correlation in echimyids appears weaker. This may be due to the relatively low size variation in our sample—particularly when *M. coypus* and capromyines are excluded—which reduces the statistical power to detect more robust allometric signals.

In many mammals, especially those with larger body sizes, increases in body and head mass are often associated with a relative shortening of the neck, a modification thought to improve mechanical stability (Arnold et al., 2017). If echimyids followed this pattern, we would expect cranio–caudal shortening of the neural arch of the atlas, as well as of the vertebral body and spinous process of the axis in larger species—features observable in the dorsal view of the atlas and the lateral view of the axis (Figure 4). In our results, however, this pattern was only subtle, becoming more apparent when comparing *M. coypus* with the markedly smaller‐bodied echimyids. Among the smaller echimyids, no consistent evidence of such shortening was detected, in agreement with previous studies on rodents and other small mammals that also failed to find significant correlations between vertebral size and neck length (Arnold et al., 2017; Vander Linden, Hedrick, et al., 2019).

It is likely that the body and head mass of most small mammals does not impose sufficient structural demands on the vertebral column—particularly the neck—to elicit such modifications, unlike in larger species that require a more robust spine to support their greater mass. Interestingly, we did observe shortening of the neural arch of the atlas and the vertebral body and spinous process of the axis in some species (e.g., *C. sulcidens*), but these features were more closely associated with semi‐fossorial locomotor modes than with size itself.

Finally, the low and marginally significant correlations between size and cervical shape in echimyids resemble patterns previously reported for their lumbar vertebrae (Netto & Tavares, 2021). This similarity may reflect shared developmental or functional constraints across vertebral regions. While we may speculate that both allometric modules are shaped by similar selective forces or morphogenetic rules, we are currently unable to disentangle the relative contributions of functional adaptation and developmental integration in generating these patterns.

### 4.2 Ecological effects

After dimensionality reduction, when PC1 and PC2 were analyzed jointly or separately, significant associations between vertebral shape and locomotor mode were identified. Significant associations were detected between vertebral shape in the four views analyzed and locomotor mode. The different views were associated with different evolutionary models, allowing different morphologies to be linked to specific habits (Tables 3 and 4). These results indicate that ecological factors, such as substrate use and locomotor behavior, played an important role in the evolution of atlas and axis morphology in echimyids. Because locomotor behaviors often co‐evolve with phylogeny, disentangling their specific effects remains challenging (Carvalhaes et al., 2022). In a recent study of small‐ to medium‐sized mammals, Taewcharoen et al. (2024) found no significant effect of locomotion on vertebral shape, particularly in small mammals, when accounting for the phylogenetic dependence of the data. However, their analysis was limited to terrestrial runners. In contrast, our findings, with a wider range of locomotor modes, suggest that ecological pressures played a key role in diversifying cervical vertebral morphology, especially in regions critical for head stabilization and mobility, such as the atlas.

LDAsbased on the first two PCs revealed substantial differentiation among locomotor modes, with classification accuracy varying across vertebral views. The caudal view of the atlas showed the highest correct reclassification rate (95.2%), whereas the lateral view of the axis showed the lowest (68.4%; Table 5). Despite the use of reduced‐dimensional data to mitigate overfitting, these results suggest that morphological differences in cervical vertebrae carry informative signals of locomotor modes. This inference is further supported by the results of evolutionary model selection, in which multi‐peak OUM frequently outperformed single‐regime models, indicating that different locomotor modes are associated with distinct adaptive optima.

Together, these findings suggest that natural selection has contributed to shaping atlas and axis morphology in echimyids, particularly in response to functional demands linked to locomotor modes. Our results differ from previous findings in Euarchontoglires, in which no significant impact of locomotor mode on atlas shape was detected (Vander Linden, Campbell, et al., 2019), but align with broader comparative analyses across mammals that identified locomotor modes as a key factor influencing cervical vertebral evolution (Vander Linden, Hedrick, et al., 2019).

Functional explanations for cervical vertebral morphology are complex, as these structures serve as attachment sites for multiple muscle groups involved in distinct biological roles—including feeding, locomotion, defensive behaviors, and posture control (Amson et al., 2015; Argot, 2003; Ercoli et al., 2017, 2020). In mammals, the atlas articulates with the occipital condyles of the skull, enabling head flexion and extension with minimal strain on the spinal cord (Crompton & Jenkins, 1973; Jones et al., 2018b). Complementarily, the odontoid process of the axis serves as a pivot, allowing for lateral rotation of the atlas and skull (Argot, 2003; Evans, 1939). Given these biomechanical functions, morphological variation in the articular facets of the atlas likely reflects differences in head mobility across species. In the following sections, we provide functional interpretations of atlas and axis morphology in relation to the locomotor modes observed among echimyids.

Arboreal taxa interact with their substrates in complex ways, likely requiring greater head mobility across multiple axes, albeit generally involving slower movements (Dunnum & Salazar‐Bravo, 2004; Emmons, 1981). Along with the semi‐aquatic *M. coypus*, these species tend to show more rounded caudal articular facets (Figure 2h). In general, wide and rounded articulations, such as those found in arboreal echimyids (Figure 4b), facilitate broader ranges of rotational movement, likely due to reduced constraints on joint mobility (e.g., Gaudioso et al., 2017).

Conversely, terrestrial echimyids tend to exhibit cranial articular facets with a pronounced medial concavity, a medio‐laterally compressed dorsal portion, and an expanded ventral section (Figure 4a). Their caudal articular facets are more triangular than those observed in other locomotor groups. These facet morphologies would likely restrict head flexion, extension, and lateral movement while enhancing stability between the skull and neck (Arlegi et al., 2022). Additionally, terrestrial echimyids, and likely the semi‐fossorial ones (Figure 2a,c), show greater ventral projection of the atlas ventral tubercle compared to arboreal and semi‐aquatic taxa (Figure 2b,d), suggesting a mechanical advantage for the muscles that insert there. This trait likely provides a broader attachment site for muscles responsible for lateral head movement, such as the longus colli muscle, which also plays a key role in cervical spine stabilization and neck flexion (Argot, 2003; Carrizo & Díaz, 2013; Combers, 2010). These muscles function as dynamic stabilizers, acting to counter axial buckling through controlled tension (Combers, 2010). This is particularly relevant for terrestrial echimyids, which exhibit high agility, including rapid movements and substantial spinal flexion, especially during predator evasion (Cerqueira et al., 1990). We hypothesize that this morphology enhances head stability during agile maneuvers. In semi‐fossorial echimyids, it may represent a further adaptation for digging by strengthening neck flexion and stabilizing the cervical region to withstand mechanical stress.

Among semi‐aquatic echimyids, the atlas typically presents a cranio‐caudally expanded neural arch and a laterally elongated yet cranio‐caudally reduced transverse process, resulting in a vertebra that is both wider and more cranio‐caudally extended (Figure 2l). This anatomical configuration is consistent with patterns observed in large‐headed or aquatic mammals and is often associated with robust necks capable of sustaining substantial loads or resisting hydrodynamic forces during swimming (Arnold et al., 2017). Notably, this condition is especially accentuated in the semi‐aquatic *M. coypus*, which exhibits the most pronounced expansion of the neural arch among the species examined. The expanded transverse processes serve as attachment sites for intervertebral ligaments and flexor muscles of the neck (Ryan, 1989). The cervical spine is under constant stress from the head's weight, creating a tendency toward ventral flexion without structural bracing (Martin et al., 1998). This stress is countered by a system of passive (ligaments) and active (muscles) elements extending from the trunk to the head. The biomechanical function of this system, particularly the nuchal elements, is to counteract the neck's bending moment (Arnold et al., 2017). The expanded transverse processes provide an enlarged attachment site not only for ligaments governing intervertebral motion but also for key cervical and pectoral muscles (Ryan, 1989). These include muscles responsible for cranial movement (e.g., obliquus capitis cranialis, obliquus capitis caudalis, rectus capitis dorsalis minor), those spanning between vertebrae (e.g., the intertransverse group), and muscles crucial for forelimb function and swimming, such as the levator scapulae and trapezius (Nalley & Grider‐Potter, 2017; Taewcharoen et al., 2025). In *M. coypus*, these structures likely provide insertion areas for enlarged pectoral girdle muscles, enhancing propulsion and maneuverability in water (Arnold et al., 2017; Vander Linden, Hedrick, et al., 2019). By increasing lever arm length and optimizing muscle orientation, this morphology would directly affect the magnitude and direction of force transmission, improving the mechanical efficiency of head and neck flexion‐extension. The reoriented transverse process may ensure that locomotor forces are not dissipated in unwanted cranial movement but are instead effectively channeled for propulsion. This suggests functional adaptations related to posture control and swimming efficiency, echoing the interpretations proposed by Slijper (1946) in his comparative work.

In the axis, semi‐aquatic and semi‐fossorial species exhibit a notably short vertebral body (Figure 2o,p), a morphology that mirrors conditions found in aquatic mammals such as cetaceans (Buchholtz, 2001) and in short‐necked digging rodents like *Ctenomys* (Ercoli et al., 2020). In aquatic taxa, the shortening of cervical elements reduces intervertebral mobility and contributes to a more streamlined body, thereby aiding efficient swimming (Buchholtz, 2001). Among semi‐fossorial rodents, particularly tooth‐ or claw‐diggers, vertebral modifications likely reflect the mechanical demands of soil displacement. These species typically adopt horizontal resting postures and display limited cervical mobility (Ercoli et al., 2020; Gambaryan et al., 2005; Hatt, 1932; Slijper, 1946). The reduction of cervical spinous processes, coupled with enlarged and vertically oriented thoracic spinous processes, increases the surface area available for the attachment of neck and dorsal musculature. These include the splenius and semispinalis cervicis—powerful neck extensors essential for lifting the head during digging activity (Carrizo & Díaz, 2013; Ercoli et al., 2020; Gambaryan et al., 2005). Among the echimyids studied, *C. sulcidens* exhibited the most accentuated version of this morphology, with a markedly shortened spinous process of the axis, consistent with its overall shortened cervical region. As proposed by VanBuren and Evans (2017), shortened or fused cervical vertebrae confer robustness and may reduce the risk of bone failure caused by powerful muscular action or repeated impacts during digging. This condition further suggests that neck dorsiflexion is a key functional component of the movement repertoire in fossorial and semi‐fossorial rodents.

### 4.3 Inference of the locomotor behavior of *E. chapalmalensis*

The fossil record of *E. chapalmalensis* is remarkably complete (Supplementary Figure S1), with nearly the entire skeleton preserved (Olivares, 2009; Olivares & Verzi, 2015). This species exhibited a distinctive craniomandibular and brain morphology compared to other echimyids, and its extinction in the Late Pleistocene has been linked to a reduction in morphological diversity within the family (Fernández Villoldo et al., 2025; Olivares et al., 2020).

In our LDA analyses, *E. chapalmalensis* was classified as semi‐fossorial or terrestrial. This inference is consistent with results from both the dorsal view of the atlas and the lateral view of the axis. The PCA of the dorsal atlas, which effectively separated locomotor modes, suggested a semi‐fossorial lifestyle. Similarly, the lateral view of the axis, which discriminated semi‐fossorial modes with greater accuracy, also placed *E. chapalmalensis* among non‐fossorial taxa.

These results align with previous studies suggesting that *Eumysops* was adapted to open environments. Its cranio‐mandibular specializations and postcranial anatomy have been interpreted as indicative of either epigean or semi‐fossorial modes (Olivares, 2009; Olivares et al., 2020). Fossils of this species are frequently found in burrows preserved in the Chapadmalal Formation (Upper‐Early to Late Pliocene) of central Argentina, in association with unequivocally fossorial taxa such as extinct ctenomyids of the genus *Actenomys* (AIO pers. obs.; Genise, 1989). Moreover, its relatively long feet and limb proportions have previously been interpreted as cursorial adaptations, possibly associated with saltatory locomotion (Horovitz, 1991; Olivares, 2009; Tavares & Pessôa, 2020).

This seemingly mixed locomotor profile finds a modern parallel in the torch‐tail rat *Trinomys yonenagae*, which inhabits dune fields in the tropical Caatinga in northeastern Brazil. Although cursorial, *T. yonenagae* excavates communal burrows used to avoid extreme temperatures (Rocha, 1995), and it exhibits asymmetrical gaits, such as transverse gallop, half‐bounds, and bounds—typical of species adapted to open habitats (Rocha, 1995; Rocha et al., 2007). Notably, *T. yonenagae* shares several anatomical and ecological traits with *Eumysops*, including aspects of cranio‐mandibular morphology, postcranial anatomy, and cervical vertebrae shape (Olivares, 2009; Olivares et al., 2020).

In environments like those potentially inhabited by *Eumysops*, it is plausible to find species that combine agile terrestrial locomotion with the ability to dig shallow burrows as refuge. As highlighted by Chen and Wilson (2015), saltatorial species often also possess digging capabilities, which can lead to ambiguous or misleading locomotor inferences when only isolated skeletal elements are analyzed. Although their study focused on fore‐ and hindlimb bones, the same caution is warranted for interpretations based solely on atlas and axis morphology.

## 5 CONCLUSIONS

This study supports recent findings indicating that the early diversification of locomotor modes in Echimyidae was pivotal to their morphological radiation, influencing external traits, cranial features, and elements of both the axial and appendicular skeletons. Our results demonstrate that the shapes of the atlas and axis in echimyids are shaped not only by ecological factors, such as locomotor mode, but also by phylogenetic relatedness. While some clades and locomotor groups exhibit overlap in morphospace, the morphology of these cervical vertebrae provides informative signals that may assist in identifying the predominant locomotor strategy and phylogenetic affiliation of echimyid taxa—particularly when dealing with incomplete or fragmentary specimens. These findings underscore the value of axial morphology, and specifically the atlas and axis, as a source of insight into the adaptive specializations of both extant and extinct species.

## AUTHOR CONTRIBUTIONS

**Thomas Furtado da Silva Netto:** Resources; data curation; software; formal analysis; writing – review and editing; visualization; validation; methodology; conceptualization; investigation; writing – original draft; project administration. **João Felipe Leal Kaiuca:** Methodology; formal analysis; writing – review and editing. **Adriana Itatí Olivares:** Conceptualization; investigation; funding acquisition; writing – original draft; writing – review and editing; visualization; methodology; validation; project administration; resources; supervision; data curation. **William Corrêa Tavares:** Conceptualization; investigation; funding acquisition; writing – original draft; writing – review and editing; visualization; validation; methodology; software; formal analysis; project administration; data curation; supervision; resources.

## Supporting information


**Supplementary Figure S1:** Atlas and axis of *Eumysops chapalmalensis* MMP 4201‐M. A, cranial view; B, caudal view, and C, dorsal view of atlas; D, lateral view of axis. Scale = 10 mm.

**Table S1.** List of examined specimens included in all analysis.

**Table S2.** List of the selected landmarks and semi‐landmarks.

**Table S3.** Results of Procrustes ANOVA.

**Supplementary Material S4:** Classification table reporting the percentage of echimid “correctly” reclassified to their original locomotor habit (left) and the inferred locomotor habits for *Eumysops chapalmalensis* with their respective posterior probabilities (right) for the atlas' caudal, cranial and dorsal views, and for the axis' lateral view. A. Results obtained with all specimens' values dataset and without phylogenetic structuring of data. B. Results from linear discriminant analyses trained on 1000 bootstrap iterations of all individuals; Percentage of posterior probabilities of assigning *Eumysops chapalmalensis* to each locomotor habit (right); the number of iterations that classified *E. chapalmalensis* to each locomotor habit (center)Training data took into account the phylogenetic structure of the data Abbreviations: SF, semifossorial.

**Table S5:** Classification table resulting from linear discriminant analyses trained on 1000 bootstrap iterations of the species average dataset for the atlas' caudal, dorsal and cranial views and for the axis' lateral view. Training data took into account the phylogenetic structure of the data. The table reports the minimum, mean and maximum values obtained in these iterations for: percentage of echimyids “correctly” reclassified to their original each locomotor habit (left); percentage of posterior probabilities of assigning Eumysops chapalmalensis to each locomotor habit (right); the number of iterations that classified E. chapalmalensis to each locomotor habit (center). Abbreviations: SF, semifossorial; Bold: higher values.
